# The relationship between the urokinase-type plasminogen activator gene Pro141Leu polymorphism and patients with prostate cancer

**DOI:** 10.1590/1806-9282.20250097

**Published:** 2025-08-08

**Authors:** Cemil Gülüm, Pelin Eroğlu, Rojda Tanrıverdi, Murat Bozlu

**Affiliations:** 1 Mersin University, Faculty of Science, Department of Chemistry – Mersin, Turkey.; 2 Mersin University, Faculty of Medicine, Department of Biochemistry – Mersin, Turkey.; 3 Mersin University, Faculty of Medicine, Department of Urology – Mersin, Turkey.

**Keywords:** Prostate cancer, Single nucleotide polymorphism, Plasminogen activator, Urokinase, Genetics

## Abstract

**OBJECTIVE:**

Although many genes associated with prostate cancer have been identified, there are still many cases of prostate cancer whose genetics have not been identified. The urokinase-type plasminogen activator gene is thought to play a key role in cancer invasion and metastasis. The Pro141Leu polymorphism may be involved in the development of prostate cancer, although genetic evidence is lacking.

**METHODS:**

We used the real time polymerase chain reaction based TaqMan assay to investigate whether the urokinase-type plasminogen activator gene Pro141Leu polymorphism is associated with prostate cancer patients and healthy controls. urokinase-type plasminogen activator, urokinase-type plasminogen activator receptor, and prostate-specific antigen antigens were assayed by the enzyme-linked immunosorbent assay method.

**RESULTS:**

We found no significant differences between the cancer and control populations in terms of genotype distribution (CC, CT, and TT). Furthermore, we found no association between the urokinase-type plasminogen activator gene Pro141Leu polymorphism and cancer risk in prostate cancer patients.

**CONCLUSION:**

This is the first study to investigate the association between the rs2227564 polymorphism and prostate cancer. Although the urokinase-type plasminogen activator gene Pro141Leu polymorphism has been reported to be a risk factor for some cancers, our findings suggest that the urokinase-type plasminogen activator gene Pro141Leu polymorphism is not an important genetic risk factor for prostate cancer.

## INTRODUCTION

According to the Global Cancer Observatory records in 2020, prostate cancer (PCa) is the second most common type of cancer in men. It is the fifth leading cause of cancer deaths. There is a higher incidence rate in developed countries due to differences in PCa diagnostic practices (e.g., the use of prostate-specific antigen [PSA])^1^. Family history and ethnicity significantly increase the risk of PCa^2^. This situation implies genetic susceptibility^3,4^.

Urokinase-type plasminogen activator gene (PLAU) is believed to play a key role in tissue degradation and cell migration under various normal and pathological conditions, including cancer invasion and metastasis. It may be responsible for the development of PCa, although there is a lack of genetic evidence^5^.

PLAU encodes a serine protease, urokinase-type plasminogen activator (uPA). uPA plays a central role during fibrinolysis. Moreover, uPA and its receptor are also involved in different biological processes such as the production of mature myeloid cells, spermatogenesis, chemotaxis, cell migration, wound healing, and mediation of different types of immune responses. Besides, it is an extracellular matrix-degrading protease that is involved in the invasiveness and progression of cancer. The oncogenic role of PLAU in several cancers has long been suggested^6^.

Because of these functions of the plasminogen activator system, the components of the plasminogen activator system are thought to be suitable targets for therapeutic approaches aimed at treating cancer or stopping its progression^7^.

Pro141Leu (rs2227564) is a genetic polymorphism. The missense mutation causes replacement of proline with leucine at position 141 of the uPA protein. The substitution of proline with leucine at position 141 could potentially alter the local structure of the uPA protein, affecting its function. This could lead to changes in protein stability, folding, and interaction with other molecules^8^.

Different findings have been reported about the relationship between the Pro141Leu polymorphism in the PLAU gene and cancer cases. There are reports in the literature that the rs2227564 polymorphism is not associated with colorectal cancer and ovarian cancer and reports that it is associated with the development of oral squamous cell carcinoma of the tongue and the invasive phenotype of gastric cancer^9,10^. No study has been conducted between PCa and the rs2227564 polymorphism to our knowledge.

The aim of this study was to determine the association of the Pro141Leu polymorphism in the PLAU gene with PCa, and also to investigate the changes in serum expression levels of uPA and its receptor (uPAR) in PCa patients compared with controls.

## METHODS

### Study group

The study included 50 patients diagnosed with PCa and 50 controls who presented to the urology department of the local university hospital between April 2022 and August 2023. Participants were aged 48–80 years and without other malignancies. Patients with a histological diagnosis of PCa by prostate biopsy were included in the study. Patients with lower urinary tract symptoms, increased prostate volume on ultrasound, PSA levels within the normal range, and no suspicious findings of malignancy on digital rectal examination were included in the control group. Participants who smoked and consumed alcohol were excluded. Participants who met the inclusion criteria were thoroughly informed about the study and signed an informed consent form.

This study received approval from the local ethics committee on March 23, 2022, under committee decision number 2022/197.

Funding was provided by the University Scientific Research Projects Unit under code 2022-2-TP3-4721.

### Urokinase-type plasminogen activator, urokinase-type plasminogen activator receptor, and total prostate-specific antigen analyses

uPA and uPAR were analyzed by a commercially available sandwich enzyme-linked immunosorbent assay (ELISA) immunoassay (BT LAB, China) according to the manufacturer's instructions. Color intensity at 450 nm was determined using an ELISA device (MultiscanGo, ThermoScientific, Finland).

Total PSA (T-PSA) levels were analyzed by the two-site immunoenzymatic (sandwich) method using a commercially available Access Hybritech PSA assay (Beckman Coulter, USA) in the UniCel DxI800 hormone autoanalyzer (Beckman Coulter, USA).

For analysis, venous blood samples were collected in tubes without contents and then spun at 2,000×*g* for 20 min. For uPA and uPAR, the serum obtained was stored at −20°C until the day of analysis. For T-PSA, the assay was performed on the day of blood collection.

### Urokinase-type plasminogen activator gene Pro141Leu (rs2227564) polymorphism analyses

Venous blood samples were collected from patients and controls in tubes containing ethylenediaminetetraacetic acid (EDTA). Whole blood samples were stored at 4°C until the day of DNA isolation. Genomic DNA was isolated using the DNA Blood Kit according to the manufacturer's protocol (Roche, Germany). DNA samples were stored at −20°C. For genotyping, a custom TaqMan genotyping assay (rs2227564) (Thermo Scientific, Lithuania) was used to determine genotypes from the isolated DNA samples using an real-time polymerase chain reaction (RT-PCR) system (LightCycler 480-II, Roche, Germany). Polymerase chain reaction (PCR) conditions were as follows: (I) pre-denaturation at 95°C for 10 min, (II) 40 cycles of denaturation at 90°C for 15 s, annealing at 60°C for 60 s, and extension at 60°C for 60 s, and (III) cooling at 40°C for 30 s.

### Statistical analysis

Continuous variables were expressed as medians with ranges, while categorical variables were expressed as frequencies and percentages. Extreme values were checked using the z-score method and removed from the dataset. Normality was checked using the Shapiro-Wilk test. The Mann-Whitney U test was used to compare the two groups. The relationships between parameters were evaluated using Spearman's correlation analysis. The association between disease and genotype was examined using the Pearson's chi-squared test. Descriptive statistics were performed using a demo version of SPSS 21 for Windows (SPSS, Chicago, IL, USA). The level of statistical significance was set at p<0.05.

## RESULTS

In this study, only males were included as patients and controls. The mean age of the control group was 62±7.36 years, and the mean age of the PCa patients was 65±7.83 years, and there was no statistically significant difference between them (p=0.054) (Table 1).

**Table 1:** Age, total prostate-specific antigen, urokinase-type plasminogen activator, and urokinase-type plasminogen activator receptor findings in prostate cancer patients and healthy volunteers.

Parameters	Patients (n=50)	Controls (n=50)	p-value^c^
Age (mean±SD^a^)	65±7.83	62±7.36	0.054
T-PSA (ng/mL) median (QR^b^)	11.80 (7.53–17.44)	1.03 (0.69–1.69)	0.001
uPA (ng/mL) median (QR^b^)	927.44 (793.34–1,049.87)	1,050.52 (916.24–1,671.52)	0.003
uPAR (ng/mL) median (QR^b^)	3.54 (3.10–3.85)	3.37 (2.99–5.02)	0.882

a Standard deviation

b quartile range (Q_25_–Q_75_)

c p-values: degree of significance between groups (p<0.05).

SD: standard deviation; T-PSA: total prostate-specific antigen; QR: quartile range; uPAR: urokinase-type plasminogen activatorreceptor.

The median T-PSA level in PCa patients was 11.80 ng/mL, with an interquartile range of 7.53–17.44 ng/mL. In the control group, the median T-PSA level was 1.03 ng/mL, and the interquartile range was 0.69–1.69 ng/mL. There was a statistically significant difference in T-PSA levels between the patient and control groups (p=0.001) (Table 1).

The median and interquartile range of uPA were 927.44 ng/mL (793.34–1,049.87 ng/mL) for the patient group and 1,050.52 ng/mL (916.24–1,671.52 ng/mL) for the control group. The median and interquartile range of uPAR were 3.54 ng/mL (3.10–3.85 ng/mL) in the patient group and 3.37 ng/mL (2.99–5.02 ng/mL) in the control group. There was a statistically significant difference between the groups for uPA levels (p=0.003) but no significant difference for uPAR (p=0.882) (Table 1).

A weak positive relationship (r=0.302) was found between age and T-PSA, a weak negative relationship (r=-0.233) between T-PSA and uPA, and a strong positive relationship (r=0.843) between uPA and uPAR (Table 2).

**Table 2:** Correlation analysis findings between age, total prostate-specific antigen, urokinase-type plasminogen activator, and urokinase-type plasminogen activator receptor.

Parameters	Age	T-PSA	uPA	uPAR
Age	1	**0.302**	-0.202	-0.111
T-PSA		1	**-0.223**	0.131
uPA			1	**0.843**
uPAR				1

Bold values significant at the 0.01 level. T-PSA: total prostate-specific antigen; uPA: urokinase-type plasminogen activator; uPAR: urokinase-type plasminogen activatorreceptor.

The genotype and allele frequency distributions of the rs2227564 polymorphism were not statistically significant between the groups (p=0.248 and p=0.121, respectively). The CC, CT, and TT genotype distributions in the PCa and control groups were as follows: 84, 16, and 0% in the PCa group and 74, 22, and 4% in the control group, respectively. In addition, the frequencies of the C and T alleles were determined to be 92 and 8% in the PCa group and 85 and 15% in the control group, respectively (Table 3).

**Table 3:** Genotype and allele frequencies of urokinase-type plasminogen activator gene Pro141Leu polymorphism.

Genotype/allele		Patients (n=50)		Controls (n=50)		p-value^a^
		N	%	n	%	
**Genotype**						
	CC	42	84	37	74	0.248
	CT	8	16	11	22	
	TT	0	0	2	4	
**Allele**						
	C	92	92	8	8	0.121
	T	85	85	15	15	

a p-values: degree of significance between groups (p<0.05).

Because the distribution of alleles according to genotype was homogeneous, the Hardy-Weinberg balance test was not used and odds ratios were not calculated.

## DISCUSSION

PCa is the second most common cancer and the fifth leading cause of cancer deaths in men in 2020. Established risk factors are limited to advancing age, family history of the malignancy, and certain genetic mutations^1^.

uPA and uPAR play a role in fibrinolysis, in various biological processes, and in the invasiveness and progression of cancer. uPA and uPAR are thought to be suitable targets for therapeutic approaches aimed at treating cancer or stopping its progression^5^.

PSA is a glycoprotein produced by the epithelial cells of the prostate. It is not specific to PCa. However, there is a significant increase in PCa^11^. As expected, the median T-PSA level in the patient group was higher than the median T-PSA level in the control group, and this was statistically significant (p=0.001). It is recommended that PSA levels increase with age and that age-related PSA levels be taken into consideration when discussing the presence of PCa or prostate-related malignancy^11^. In our study, T-PSA levels showed a positive correlation with age (r=0.302).

Increased expression of uPA has been reported in several malignancies, including PCa^12,13^. In this study, we found no statistically significant difference between serum uPAR levels in PCa patients compared to controls (p=0.882). However, low serum levels of uPA were statistically significantly different in PCa patients compared to controls (p=0.003). In their study, Miyake et al.^14^ reported an increase in uPA and uPAR expression in the PCa patient group compared to the control group, in contrast to our results (p=0.05 for uPA and p=0.005 for uPAR). Milanese et al.^15^ also reported in their study that uPAR expression increased statistically significantly in the patient group (p≤0.0001). Serafin et al.^16^ reported that the uPA was significantly lower in PCa patients compared to controls (p<0.0001). This may be due to the inability of ELISAs to distinguish between the different forms of uPA. Lei et al.^17^ reported that measuring the components of the uPA system in plasma by ELISA generally gives erroneous results. We believe that diagnostic and therapeutic approaches based on uPA and uPAR indices should be approached with greater skepticism due to conflicting evidence.

The etiology of PCa remains unclear. In recent decades, many researchers have drawn attention to the association between genetic polymorphisms and cancer susceptibility. Indeed, a significant number of studies have identified numerous single-nucleotide polymorphisms (SNPs) that contribute to the incidence of cancer, including PCa.

The present study investigated the PLAU polymorphism in PCa and found no association between the Pro141Leu polymorphism and PCa risk. Patients diagnosed with PCa exhibited C and T allele frequencies of 92 and 8%, respectively. However, these findings were not statistically significant between the control and patient groups (p=0.121). According to data from studies conducted in different regions and populations in the National Library of Medicine, the frequency of the C allele in the rs2227564 SNP was found to be 78%, and the frequency of the T allele was found to be 22% worldwide. In their study, Savas et al.^18^ reported that the C allele was present in 97.9% of Africans, 78.3% of Asians, and 68.8% of Caucasians. In contrast, the T allele was present in 2.1, 21.7, and 31.2% of the aforementioned groups, respectively. To our knowledge, this is the first study to investigate the association between the rs2227564 polymorphism and PCa.

Furthermore, studies investigating the association between the rs2227564 polymorphism and other cancers have reported different results. Wu et al.^19^ reported that the rs2227564 polymorphism is associated with the invasive phenotype of gastric cancer. Bentov et al.^20^ reported that this polymorphism is not associated with ovarian cancer risk. No association between colorectal cancer and the rs2227564 SNP was reported by Försti et al^21^. Duran et al.^22^ reported that patients with the Pro141L T allele variant have a higher risk of developing poor coronary collateral circulation. In the study conducted by Zhong et al.^10^, it was reported that the rs2227564 polymorphism is associated with the development of oral tongue squamous cell carcinoma.

In their meta-analysis study investigating the relationship between polymorphisms of the urokinase plasminogen activator system and cancer, Xu et al.^9^ reported that there was no significant relationship between the rs2227564 polymorphism and cancer risk. However, they also stated that large-scale case–control studies are necessary to determine the accurate effects of the rs2227564 polymorphism on cancer development.

## CONCLUSION

The present study in the Turkish population found that the Pro141Leu polymorphism was not associated with the development of PCa. Thus, although the PLAU Pro141Leu polymorphism has been reported to be a risk factor for some cancers, it does not appear to play a major role in the development of PCa, at least in the Turkish population. Of course, large case–control studies are needed to determine the exact effect of the rs2227564 polymorphism on PCa development.

## Data Availability

The datasets generated and/or analyzed during the current study are available from the corresponding author upon reasonable request.
